# Highly Efficient Cationic/Anionic Cellulose Membranes for Removal of Cr(VI) and Pb(II) Ions

**DOI:** 10.3390/membranes13070651

**Published:** 2023-07-06

**Authors:** Lu Liu, Hongyang Ma, Madani Khan, Benjamin S. Hsiao

**Affiliations:** 1 State Key Laboratory of Organic-Inorganic Composites, Beijing University of Chemical Technology, Beijing 100029, China; 2 Department of Chemistry, Stony Brook University, Stony Brook, NY 11794-3400, USAbenjamin.hsiao@stonybrook.edu (B.S.H.)

**Keywords:** cationic/anionic cellulose membrane, oxidation, adsorption, heavy metal ions, wastewater treatment

## Abstract

To achieve high throughput, low-pressure drops, and high adsorption capacity of Cr(VI) and Pb(II) in industrial wastewater treatment, cellulose membranes containing cationic and anionic groups were fabricated, respectively. In this process, cost-effective cotton fabrics were oxidized using sodium periodate, followed by quaternary ammonium or sulfonation modifications. The chemical composition, surface morphology, and thermal and mechanical properties of the cellulose membranes were investigated by ATR-FTIR, solid-state NMR, SEM, TGA, and tensile experiments. Quaternary ammonium, aldehyde, and sulfonate groups were distributed on the cationic/anionic cellulose fibers as adsorption sites, which issue remarkable adsorption capability to the cellulose membranes. The highly toxic Cr(VI) and Pb(II) ions were used to challenge the adsorption capacity of the cationic and anionic cellulose membranes, respectively. The maximum adsorption capacities of Cr(VI) and Pb(II) ions were 61.7 and 63.7 mg/g, respectively, suggested by Langmuir isotherms, kinetics, and thermodynamics in the static experiments. The dynamic adsorption capability of cationic cellulose membranes against Cr(VI) ions was determined and compared with that of commercially available anionic-exchange membranes. Spiral wound filtration cartridges were fabricated by cationic and anionic cellulose membranes, respectively, and were used to adsorb Cr(VI) and Pb(II) from lab-made wastewater, respectively. The cationic cellulose cartridge can purify 4.4 L of wastewater containing 1.0 mg/L of Cr(VI) ions with a 100% removal ratio, while the pressure drop was retained at 246 Pa. Similarly, the anionic cellulose cartridge exhibited even more impressive adsorption capability; the removal ratio against Pb(II) was 99% when 8.6 L of 1.0 mg/L of Pb(II) ions containing wastewater was treated, and the pressure drop was retained at 234 Pa. A composite cartridge fabricated by the integration of cationic and anionic cellulose membranes was successfully employed to purify the wastewater containing Cr(VI) and Pb(II) simultaneously. The possible adsorption mechanism was proposed, and the recycling ability of the cellulose membranes was also discussed.

## 1. Introduction

Wastewater containing heavy metals such as chromium (Cr(VI)), lead (Pb(II)), mercury (Hg (II)), and arsenic (As (III)) [1,2,3,4] causes a serious threat to the ecological environment and human health; therefore, the removal of heavy metal ions from wastewater is a great concern regarding their toxicity at trace levels and enrichment in biological systems. It is worth noting that Cr(VI) is listed at the top of the priority of toxic pollutants by the US EPA, and the contents of Cr(VI) and Pb(II) in drinking water should not exceed 0.05 and 0.01 mg/L [5], respectively. In the recent past, various approaches including chemical precipitation, adsorption, membrane filtration, electrolysis, coagulation, and flocculation [6,7,8,9] have been employed for removing heavy metal ions; however, they suffer the limitations such as secondary contamination, low removal capacity and selectivity, and cost-effectiveness. Among these methods, adsorption exhibits more advantages over others for the removal of heavy metal ions and has attracted more attention as high adsorption capacity, high selectivity, and high removal efficiency were simultaneously achieved [10]. On the other hand, biodegradable materials such as cellulose have original advantages in terms of high functionality, unique physicochemical properties, environmental friendliness, and cost-effectiveness. As a result, nanoscaled cellulose was employed as a highly efficient adsorbent based on various surface modifications with nanocellulose [11,12], polyaniline [13,14], and polypyrrole [15,16]. For example, Xu et al. oxidized nanocellulose with sodium periodate and then reacted the dialdehyde nanocellulose product with activated hydrogen on black vitrine tannin. The obtained material showed good adsorption capacity for Pb(II) (55.6 mg/g), which could be recycled more than five times and could successfully eliminate a variety of heavy metal ions, for example, Cu(II), Pb(II), and Cr(VI). However, for the adsorption of Pb(II), it has a slow rate of adsorption (4 h to reach equilibrium), and there is a lack of studies on the interference of coexistence ions within the same sample [17]. In another study, the removal capability of Cr(VI) from wastewater was investigated using a combination of nanocellulose and quaternary ammonium groups. In this process, sugarcane bagasse cellulose was chosen after periodate oxidation followed by grafting with Girard’s reagent T (GT) containing quaternary ammonium groups. A high adsorption capacity of 80.45 mg/g of the cationic nanocellulose was achieved successfully at pH 6.0, indicating that GT introduced effectively positive adsorption sites for Cr(VI) adsorption. However, the competing ions such as Cl^−^, NO_3_^−^, and SO_4_^2−^ drastically affected the adsorption capacities of the cationic nanocellulose against Cr(VI) when the concentration of the competing ions increased to 1000 ppm. Meanwhile, the cationic nanocellulose was used only in the suspension, which needs additional separation after adsorption. Moreover, it was found that the aldehyde groups (about 75% were left over) were not substituted; thus, they did not help with the adsorption [18]. Therefore, despite their remarkable adsorption capability to various pollutants, modified nanocellulose adsorbents still present some challenges in post-treatment, effectiveness of functional groups, and adsorption selectivity.

Sustainable carboxylated cellulose fabrics with remarkable mechanical properties were fabricated by TEMPO-mediated oxidation and were directly employed as highly efficient filters for the removal of methylene blue (MB) and Pb(II) as well as recycling of lanthanum by dynamic adsorption, which required no post-treatments. It was found that the adsorption capacities of optimized cellulose fabrics oxidized by 4.0 mmol/g of sodium hypochlorite were higher than that of most adsorbent counterparts. The high adsorption capability was due to the functionalization inside of the cellulose fibers and was less affected by competing ions such as sodium, potassium, and calcium ions even with 2~5 times higher concentrations. This inspired us to use cellulose fabrics for the development of highly efficient membranes for Cr(VI) and Pb(II) adsorption [19].

For this study, quaternized ammonium and sulfonated cellulose membranes were fabricated by periodate oxidation, followed by grafting of GT and sulfonation. The membranes were employed to remove Cr(VI) and Pb(II) ions, respectively. Quaternary ammonium and sulfonic acid groups served as adsorption sites based on those ions via electrostatic interactions. The adsorption kinetics, isotherms, and thermodynamics of Cr(VI) and Pb(II) on quaternized and sulfonated cellulose membranes were investigated comprehensively, and a multifunctional adsorption mechanism was proposed. Meanwhile, the effects of competing ions on the adsorption capacity of the quaternized cellulose were studied. Spiral wound filtration cartridges based on modified cellulose membranes were assembled, and the dynamic adsorption of Cr(VI) and Pb(II) ions was demonstrated. The recyclability of cationic/anionic cellulose membranes was also evaluated.

## 2. Materials and Methods

### 2.1. Reagents

Cotton fabrics were purchased from Shaoxing Xiangjia Textile Co., Ltd., Shaoxing, China, and pretreated in a boiling water bath for 15 min before use. Sodium periodate (AR), potassium dichromate (AR), and sodium bisulfite (AR) were bought from Macklin (Shanghai, China), while Girard’s reagent T (GT, 98%) and diphenyl semicarbazide (AR) were purchased from Aladdin (Shanghai, China) and used as received. All other chemicals were provided by Beijing Chemical Works, Beijing, without further treatment.

### 2.2. Oxidation of Cellulose Fabrics by Periodate

Cellulose fabrics, sodium periodate, and water were weighed based on the weight ratios of 1:2.67:100, and then placed onto a 1000 mL beaker for five different samples. The mixtures were stirred at 25 °C in the dark for a pre-determined time, e.g., 3, 6, 9, 12, and 24 h, respectively. Ethylene glycol with 1.5 times the mass of cellulose fabrics was added to terminate the reaction. The oxidized cellulose fabrics were then washed completely with pure water until the conductivity of the washing solution was lower than 10 μS/cm. The samples, named DAC-3, DAC-6, DAC-9, DAC-12, and DAC-24, were dried in an oven at 50 °C for 24 h before use.

### 2.3. Surface Grafting of DACs with GT

The preparation of cationic DAC was carried out by Schiff base reaction between DAC and GT. In detail, 3.0 g of GT was dissolved in 100 mL of water in a 250 mL beaker, followed by adding 1.0 g of DAC and adjusting the pH of the mixture to 4.5. The reaction system was stirred in a water bath at 60 °C for 24 h. The cationic DAC fabrics were then washed with pure water and stopped until the conductivity of the washing solution was less than 10 μS/cm, before drying in an oven at 50 °C for 24 h. The obtained samples were named DAC-3-GT, DAC-6-GT, DAC-9-GT, DAC-12-GT, and DAC-24-GT, respectively.

### 2.4. Surface Sulfonation of DACs with Sodium Bisulfite

The anionic cellulose membrane was prepared by a sulfonation reaction between DAC and sodium bisulfite. In a 250 mL beaker, sodium bisulfite (5.0 g) was dissolved in 100 mL of water and then DAC (1.0 g) was added. The reaction system was stirred at room temperature for 24 h, followed by washing with pure water until the conductivity was lower than 10 μS/cm. The product was then placed in an oven at 50 °C and dried for 24 h. The obtained samples were named DAC-3-S, DAC-6-S, DAC-9-S, DAC-12-S, and DAC-24-S, respectively.

### 2.5. Determination of Degrees of Oxidation, Quaternization, and Sulfonation

The content of aldehyde groups in DAC samples was determined by titration method. In detail, 0.1 g of all DAC samples were chopped and mixed with 50 mL of de-ionized (DI) water, which then adjusted the pH value to 4.0. A hydroxylamine hydrochloride solution (0.72 mol/L, 5 mL) was added dropwise, and the mixture was titrated with 0.1 (or 0.5) mol/L of NaOH solution until the pH value of the system remained at 4.0 without further changes. The amount of NaOH aqueous solution consumed was recorded, and the content of aldehyde groups was calculated based on Equation (1):

(1) $$C_{-CHO}=\frac{\left(V_{2}-V_{1}\right)\times C_{NaOH}}{m}$$

where, *C_-CHO_* is the content of aldehyde groups, in mmol/g; *V*_1_ and *V*_2_ are the volumes of NaOH solution before and after titration, in mL; *C_NaOH_* is the concentration of NaOH solution, in mol/L; m is the mass of the DAC sample used for titration, in grams.

The degree of quaternization and sulfonation was determined by elemental analysis (vairo EL CUBE model, Germany) to measure the contents of nitrogen and sulfur in the samples, respectively. 

### 2.6. Characterization of Modified Cellulose Membranes

The morphologies of cellulose membranes before and after modifications were observed with a Schottky field-emitted scanning electron microscope (SEM). The samples were prepared by sputtering with platinum before observation. A Fourier transform infrared spectrometer (FTIR, Nicolet 6700, Beijing, China, Thermo Fisher Technologies LTD) was used tocharacterize the surface chemistry of the samples with an ATR model, and the range of wavenumbers was 4000–550 cm^−1^ with a resolution of 4 cm^−1^. The thermal stability of the samples was determined by a synchronous thermal analyzer (TGA/DSC3+, Zurich, Switzerland) at a temperature range of 25 °C to 800 °C with a heating rate of 10 °C/min in a nitrogen atmosphere. 

The hydrophilicity of cellulose-based membranes was investigated by the water contact angle by dropping water droplets on the sample surface (TBU goE). The changes in the water contact angles vs. time were recorded at the rate of one frame per second. 

X-ray photoelectron spectroscopy (XPS, Thermo Scientific K-Alpha, Waltham, MA, USA) was used to analyze the surface chemical composition of the samples, and the test was conducted using a single-color Al target. The XPS spectra were analyzed using XPS PEAK 41 software. X-ray diffraction profiles of the cellulose membranes were obtained with an X-ray diffractometer (XRD, Bruker D8 ADVANCE, Rigaku Corporation, Akishima, Japan). The instrument uses a Cu target ceramic X-ray tube with 2θ data scanning range of 10~50° and a scanning speed of 5°/min. The crystallinity index CrI58 was calculated based on Equation (2):

(2) $$CrI=\frac{I_{002}-I_{am}}{I_{002}}\times 100\%$$

where *I*_002_ is the maximum diffraction intensity of lattice plane (002), and *I_am_* is the diffraction intensity of the amorphous peak of cellulose samples, i.e., the minimum height between the plane (101) and lattice plane (10ī).

Tensile experiments were carried out through a universal stretching machine (CMT6103 manufacturer MTS Metus Industrial System). The samples were cut into dumbbell-type samples of 50 mm × 4 mm, and the stress–strain curves of the cellulose membranes were obtained at the tensile speed of 20 mm/min. 

A high-resolution solid-state ^13^C NMR (Brucker 400M, Karlsruhe, Germany) was employed to characterize the chemical structure before and after the surface modification of cellulose samples. The samples were ground into powder, dried, and loaded in the NMR tube (MAS rotation rate: 10 kHz); ^13^C NMR spectra were obtained after 2000 scans at 25 °C.

### 2.7. Static Adsorption

The static adsorption was carried out in batches. Typically, 10 mg of DAC-12-GT or DAC-12-S was added into a 20 mL glass vial. The glass vial contained 10 mL of Cr(VI) or Pb(II) ion solution with a pre-determined concentration and pH value. The cellulose membranes were removed from the solution after a period of adsorption, and the concentrations of the leftover solutions were determined by UV-vis spectroscopy at 540 nm and 523 nm, respectively, using 1,5-diphenylcarbamide and PAR as the staining agents, respectively. The adsorption capacities of DAC-12-GT and DAC-12-S against Cr(VI) and Pb(II) ions, respectively, were calculated according to Equation (3):

(3) $$q_{t}=\frac{(C_{0}-C_{t})\times V}{m}$$

where *q_t_* is the adsorption capacity at time *t*, in mg/g; *C*_0_ is the initial concentration of the solution, and *C_t_* is the concentration of the solution at time *t*, in mg/L; *m* is the mass of cellulose membranes, in grams.

### 2.8. Dynamic Adsorption Properties of Cationic/Anionic Cellulose Membranes

A sample disc with a diameter of 25 mm was latched in a stainless-steel filtration cell. A 50 mL syringe containing 50 mL of heavy metal solution was loaded on a syringe pump. The concentrations of the heavy metal solutions for Cr(VI) and Pb(II) ions were 1.0 and 0.1 mg/L, respectively. The pH values of the solutions containing Cr(VI) and Pb(II) were 2.0 and 5.0, respectively, being adjusted by HCl or NaOH aqueous solution. The flow rate of the aqueous solution was fixed at 0.05 mL/min. A breakthrough curve was obtained by plotting the concentration vs. volume of the permeate.

### 2.9. Influence of Coexisting Ions on Dynamic Adsorption Capacity

Using Cl^−^, SO_4_^2−^, and PO_4_^3−^ as the representatives of monovalent, divalent, and trivalent competing anions in the aqueous solution containing Cr(VI), respectively, the effect of coexisting ions on the adsorption of Cr(VI) by DAC-12-GT membrane in a dynamic adsorption process was explored. In detail, 0.824 mg of sodium chloride, 0.740 mg of sodium sulfate, and 1.176 mg of sodium phosphate were dissolved in 100 mL of Cr(VI) aqueous solution (pH = 2), separately, to obtain aqueous solutions with the concentrations of 5 mg/L of Cl^−^, SO_4_^2−^, and PO_4_^3−^, respectively. 

Similarly, sodium and zinc ions were employed as competing monovalent and divalent ions, respectively, for Pb(II) adsorption by DAC-12-S membrane. Specifically, 0.25 g of AlCl_3_, 0.20 g of MgCl_2_, and 0.08 g of NaCl were dissolved in 100 mL of Pb(II) aqueous solution (pH = 5), separately, and the final concentrations were 0.5 mg/L. Following the same protocols as the dynamic adsorption, the adsorption selectivity of cationic/anionic cellulose membranes against Cr(VI) and Pb(II) ions was investigated. The concentrations of Cr(VI) and Pb(II) ions in the permeate were determined by UV and an Inductively Coupled Plasma Emission spectrometer (ICP), respectively.

### 2.10. Adsorption Performance of SPIRAL Wound Filtration Cartridges Based on Cationic/Anionic Cellulose Membranes

Spiral wound filtration cartridges were fabricated using cellulose membranes DAC-12-GT and DAC-12-S, separately, and their long-term adsorption performance was evaluated by using 0.1 mg/L of Cr(VI) (pH = 2) and Pb(II) (pH = 5) solutions, respectively. The effective filtration area of the membrane (~5.0 g) was 360 cm^2^. The pressure drop monitored in the adsorption process was finally retained at 246 Pa and 234 Pa, respectively, while the flow rate was 1.0 mL/min. Adsorption data were recorded every 1 h, and a breakthrough curve was plotted after the demonstration. Finally, a spiral wound filtration cartridge was prepared with the combination of DAC-12-GT and DAC-12-S membranes, and the long-term adsorption performance with the aqueous solution containing both 1.0 mg/L of Cr(VI) and Pb(II) was also evaluated.

### 2.11. Desorption

The DAC-12-GT and DAC-12-S membranes after adsorption of Cr(VI) and Pb(II) were desorbed by different approaches to learning the recyclability of the membranes. A variety of desorption agents such as 1.0 wt% and 10 wt% of NaOH aqueous solutions as well as the mixture of 1 wt% of NaOH and NaCl, respectively, were prepared to remove adsorbed Cr(VI) ions. The membrane was regenerated by soaking in different desorption agents and then shaking in an oscillator (frequency 200 times/min) for 3 h. The desorbed cellulose membrane DAC-12-GT was then washed completely with pure water and dried in an oven at 50 °C for 24 h for the next adsorption. The desorption efficiency was evaluated based on Equation (4):

(4) $$Desorption\;efficiency=\frac{q_{s}}{q_{r}}\times 100\%$$

where *q_s_* and *q_r_* are the desorbed and adsorbed Cr(VI), respectively, and the unit is mg/g. The regeneration of the membrane DAC-12-S followed the same protocols except the adsorption agents were 1.0 mol/L of HNO_3_ and EDTA, respectively.

## 3. Results and Discussion

### 3.1. Oxidation, Quaternization, and Sulfonation of Cellulose Fabrics

**Figure 1:**
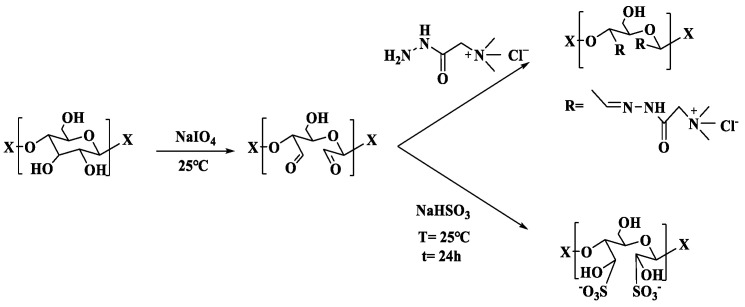
Fabrication of oxidized, quaternized, and sulfonated cellulose membranes.

Cellulose fabrics were oxidized by sodium periodate, whereby two aldehyde groups were converted from C2 and C3-hydroxymethyne groups located on cellulose, as shown in Figure 1.

The oxidized cellulose membranes were further grafted with GT and sodium bisulfite, respectively, and quaternary ammonium groups and sulfonic acid groups were introduced through Schiff base reaction and addition reaction, respectively, whereby cellulose membranes containing functional adsorption sites were generated for wastewater treatment.

**Figure 2:**
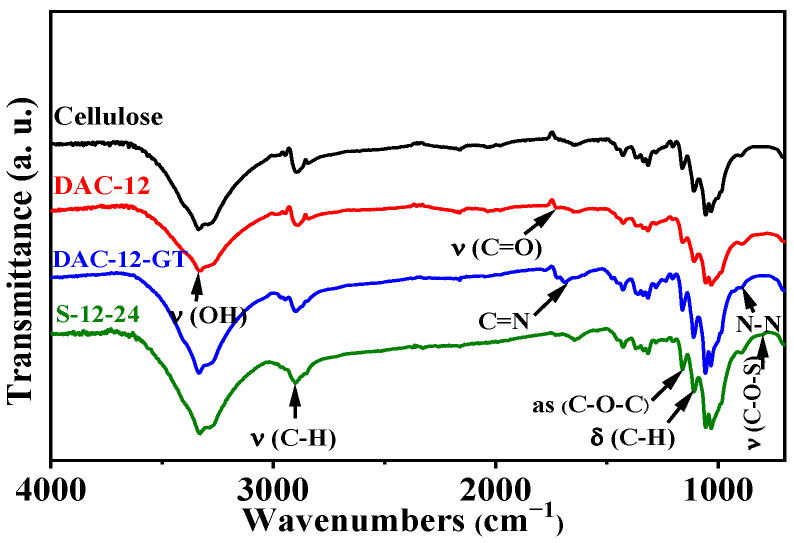
ATR-FTIR spectra of cellulose fabrics and oxidized, quaternized, and sulfonated cellulose membranes.

ATR-FTIR spectra of original cellulose oxidized, quaternized, and sulfonated cellulose membranes were obtained, as shown in Figure 2, to verify the accomplishments of the oxidation, Schiff base reaction, and addition reaction of cellulose.

There are vibration peaks [20] assigned to cellulose in all four spectra, which are 3332 cm^−1^ [υ (O-H)], 2895 cm^−1^ [υ (C-H)], 1162 cm^−1^ [υ (C-O-C, as)], and the bending vibration peaks of O-H at 1335 cm^−1^ and 1365 cm^−1^, respectively. 

Compared with cellulose fabrics, a shoulder peak at 1721 cm^−1^ in the spectrum of DAC-12 appeared, which corresponds to the stretching vibration peak [υ (C=O)] of the aldehyde groups [21], evidenced by the successful oxidation of the cellulose fabrics by periodate.

A sharp peak at 1689 cm^−1^ and weak vibration peak at 923 cm^−1^ in the spectrum of DAC-12-GT were observed, which are the imine bond and N-N bond [20], respectively, created by the reaction between the aldehyde group and amino group in GT. This offers strong evidence for the successful quaternization of DAC-12 by Schiff base reaction.

In the spectrum of DAC-12-S, the stretching vibration peak of C-O-S in –SO_3_^−^ appears at 807 cm^−1^, which proves that the sulfonation reaction of DAC-12 has successfully occurred [22], which is also supported by the results of elemental analysis.

**Figure 3:**
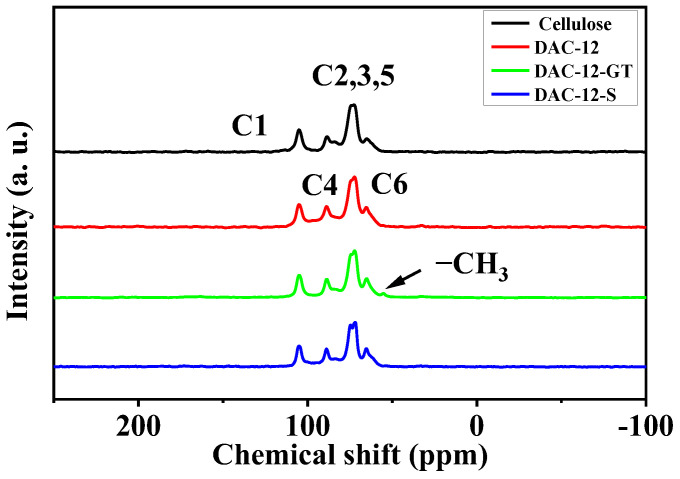
Solid-state 13C NMR spectra of cellulose fabrics and oxidized, quaternized, and sulfonated cellulose membranes.

The chemical structure of the cellulose fabrics before and after modification was also verified by solid-state ^13^C NMR, as shown in Figure 3.

It was clear that resonance peaks located at chemical shifts at 105 ppm, 89 ppm, and 65 ppm in the spectra belong to C1, C4, and C6 of cellulose structure, respectively; while the signal located in the overlap region between 71.8 and 75.4 ppm comes from C2, C3, and C5 in cellulose [23]. There is no obvious peak assigned to the aldehyde in the spectrum of DAC-12 though, and the new peak at 55 ppm in the spectrum of DAC-12-GT appeared clearly, which could be attributed to the methyl of the quaternary ammonium groups in GT [18], indicating that the grafting reaction was successful. In addition, the resonance peak assigned to C2, 3, 5 in the spectrum of DAC-12-S splits, compared to that of pristine cellulose, which implied that the sulfonating reaction probably occurred. Therefore, the solid-state NMR results verified once again that the modification of cellulose fabrics was carried out successfully.

**Figure 4:**
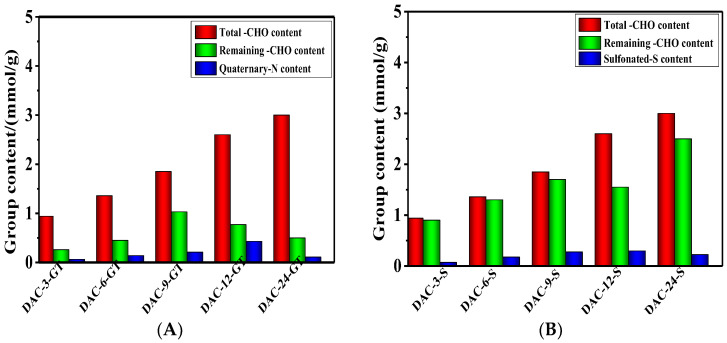
Functional group content of quaternized (**A**) and sulfonated (**B**) cellulose membranes vs. oxidation reaction time, respectively.

The quantitative content of aldehyde, quaternary ammonium, and sulfonate groups after modifications was determined by titration and elemental analysis, respectively, as shown in Figure 4.

The oxidation degree of the cellulose membrane could be adjusted by oxidation time, while the highest aldehyde content of the oxidized cellulose DAC-24 was 3.77 mmol/g. However, considering the mechanical properties of the cellulose membrane containing 3.77 mmol/g of aldehyde groups were relatively poor, the membrane with 2.60 mmol/g of aldehyde prepared by 12-h oxidation was regarded as the optimized membrane for the next modifications, i.e., quaternary and sulfonation. 

The quaternary ammonium groups were introduced by the Schiff base reaction between the amino groups of GT and the aldehyde groups of oxidized cellulose. The quaternization degree of the cellulose membrane was increased with the increasing content of aldehyde groups as expected, except for the sample DAC-24-GT which exhibited a relatively lower quaternization degree. The possible reason was that the oxidized cellulose with a high quaternization degree could be partially dissolved in the aqueous reaction system, and therefore, the leftover with a lower quaternization degree was collected. A similar trend was observed for the sulfonation of cellulose where the highest sulfonation degree was achieved when the aldehyde content of oxidized cellulose was 2.60 mmol/g. As a result, DAC-12-GT and DAC-12-S exhibited reasonably high mechanical strength and were regarded as the optimized cationic/anionic cellulose membranes. The contents of quaternary ammonium and sulfonate groups of DAC-12-GT and DAC-12-S were 0.43 and 0.30 mmol/g, respectively, measured from nitrogen and sulfur content by elemental analysis. 

The content of aldehyde groups of the cellulose membranes after surface modifications was also determined to understand the grafting reactions. The aldehyde groups of the modified cellulose were still available due to the limited access to the oxidized cellulose for the grafting reagents. However, those leftover aldehyde groups could also be used for further adsorption, e.g., the components containing amino nitrogen.

**Figure 5:**
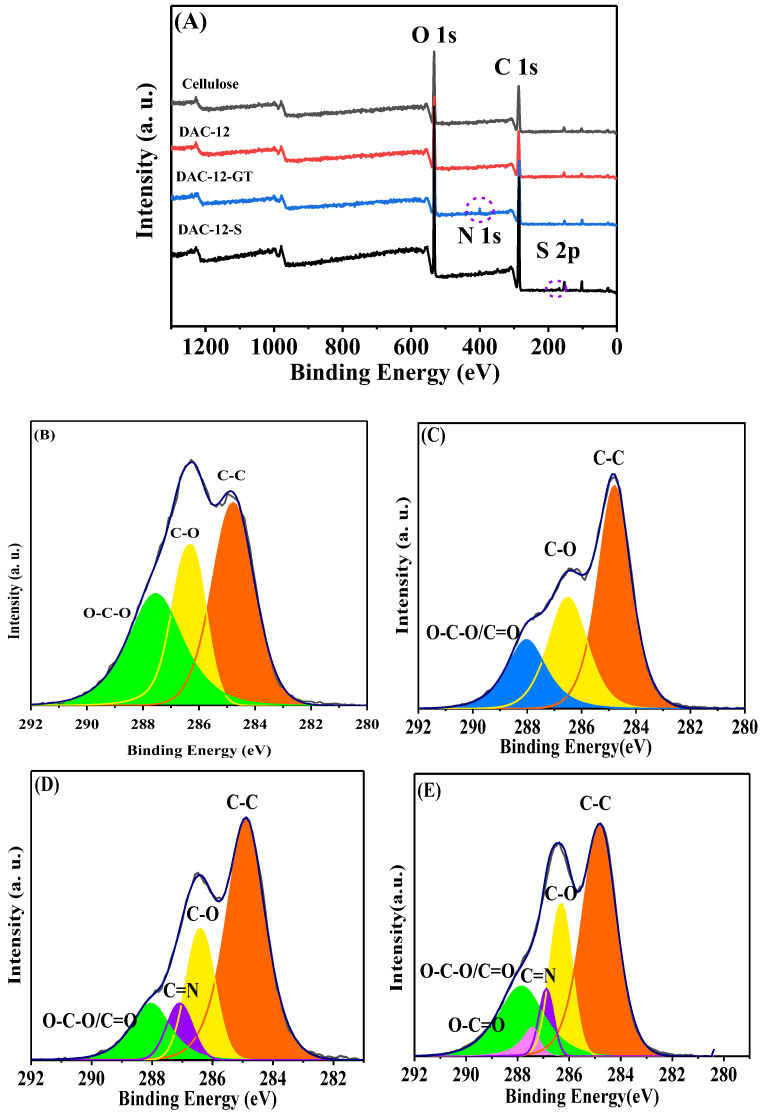
XPS spectra of wide scan (**A**) and high resolution of pristine cellulose (**B**), DAC-12 (**C**), DAC-12-GT (**D**), and DAC-12-S (**E**), respectively.

The surface chemical composition of the pristine cellulose, DAC-12, DAC-12-GT, and DAC-12-S was investigated by XPS, and wide-scan and high-resolution spectra are shown in Figure 5.

In addition to the peaks located at 286.7 and 531.9 eV, assigned to C1s and O1s in the spectra of pristine cellulose and DAC-12, there was a peak at about 400 eV in the spectrum of DAC-12-GT, which could be assigned to N1s from the GT groups, indicating GT was successfully grafted onto cellulose through Schiff base reaction. Meanwhile, the peak at 168.2 eV, corresponding to S2p, can be seen in the spectrum of DAC-12-S, which could also be attributed to the successful sulfonation of DAC-12. 

The high-resolution XPS energy spectrum peak of the C atom is divided into different binding forms (as shown in Figure 5B), i.e., C-C (284.8 eV), C-O (286.6 eV), and O-C-O (288.0 eV) [24,25]. In a high-resolution spectrum of DAC-12 membrane, a peak assigned to C=O was merged with one to O-C-O, when compared with that of pristine cellulose. However, the C=N peak at 287 eV in the spectrum of DAC-12-GT could be decoupled clearly and the content of C=N could be figured out as 0.32 mmol/g, which matched well the titration results. Similarly, the sulfonation degree could also be evaluated from the content of C-S bond, which was 0.21 mmol/g, also in agreement with the titration results. Therefore, the XPS results quantitatively confirmed the successful modification of pristine cellulose by surface quaternization and sulfonation, respectively. 

**Figure 6:**
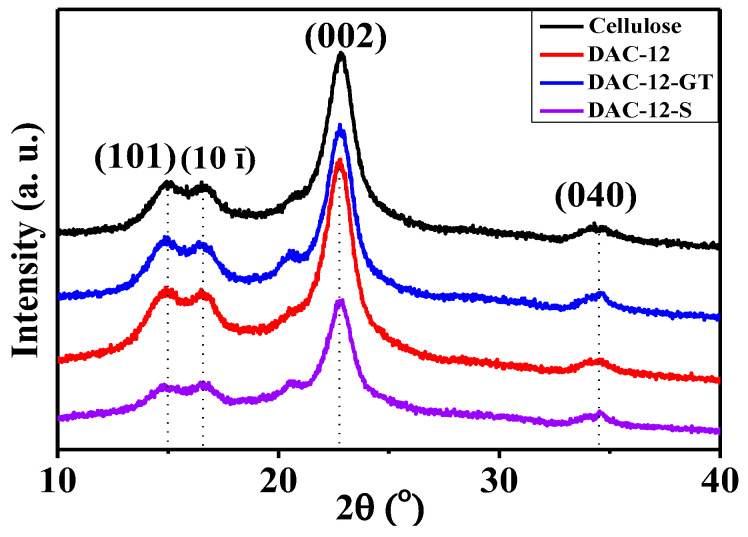
XRD patterns of cellulose fabrics before and after modification.

The crystal structure of cellulose fabrics before and after modification was analyzed regarding the changes in crystal type and crystallinity, as shown in Figure 6.

The diffraction peaks of the cellulose membranes before and after modifications are located at 15.0°, 16.6°, 22.8°, and 34.5°, respectively, which were assigned to crystal planes (101), (10ī), (002), and (040), respectively. Those diffraction peaks correspond to the structure of cellulose type I; therefore, sodium periodate oxidation, quaternization, and sulfonation exhibited fewer effects on the crystalline structure of native cellulose.

The crystallinity of cellulose membranes, i.e., DAC-12, DAC-12-GT, and DAC-12-S, was calculated based on XRD profiles. The crystallinity decreased from 62.9% to 56.1% after periodate oxidation, which probably was due to the cleavage of the chemical bond at the C2-C3 position, leading to the opening of the pyran ring and the partial destruction of the ordered structure of the cellulose molecules [26]. However, no significant changes occurred in the crystallinity when compared with that of the pristine cellulose membrane, indicating that the periodate oxidation mainly occurred in the amorphous region and the boundary of the crystal region. Moreover, the crystallinity of DAC-12-GT and DAC-12-S was 57.5% and 57.2%, respectively, which was quite similar to the 56.1% of DAC-12; unsurprisingly, the quaternization and sulfonation were only related to aldehyde groups without changing the crystal structure of the cellulose membrane [27].

### 3.2. Surface Morphology of Cellulose Membrane after Modifications

**Figure 7:**
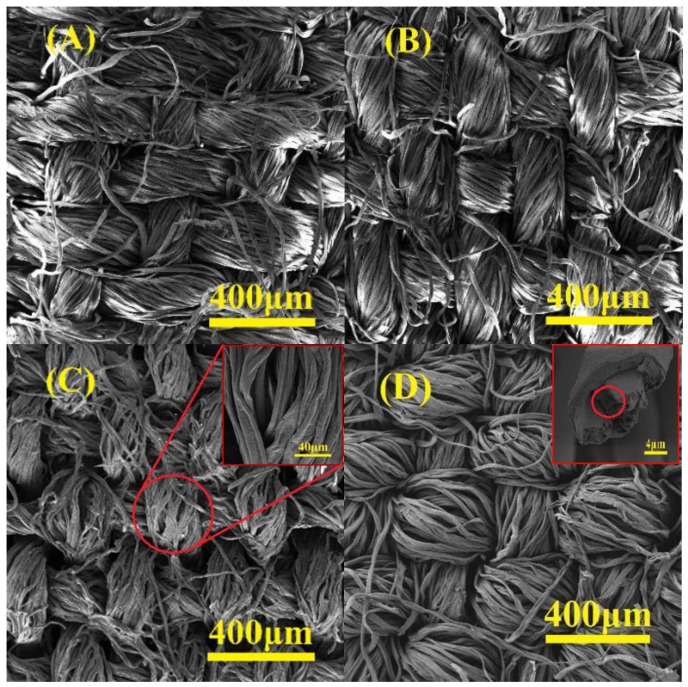
SEM images of cellulose fabrics (**A**), DAC-12 (**B**), DAC-12-GT (**C**), and DAC-12-S (**D**).

The surface morphology of cellulose membrane after oxidation, quaternization, and sulfonation was observed by SEM measurements and compared with that of pristine cellulose fabrics, as shown in Figure 7.

The surface morphology of the cellulose membrane exhibited negligible change before and after oxidation, as shown in Figure 7A, when the oxidation degree was controlled at 2.60 mmol/g, implying that the oxidation mainly occurred on the fiber surface and amorphous regions without remarkably affecting the fiber skeleton. However, significant differences can be seen after the quaternization of cellulose fabrics, as shown in Figure 7C; both rough surface and curly fiber were observed. The fiber surface became wrinkled and some of them were even broken, though the woven structure remained. Moreover, the surface morphology of the sulfonated cellulose membrane was also changed mildly, and the porous structure inside the fiber could be seen in the cross-sectional view, as shown in Figure 7D. This could be attributed to the “etching” inside the fiber by the sulfonation reaction, which was favorable to increasing the specific surface area and, therefore, the adsorption capability of modified cellulose membrane against heavy metal ions. Therefore, it was expected that the oxidation, quaternization, and sulfonation would affect the properties of cellulose membranes, e.g., thermal, mechanical, and hydrophilic properties, as well.

### 3.3. Thermal Stability of Pristine, Oxidized, Cationic/Anionic Cellulose Membranes

**Figure 8:**
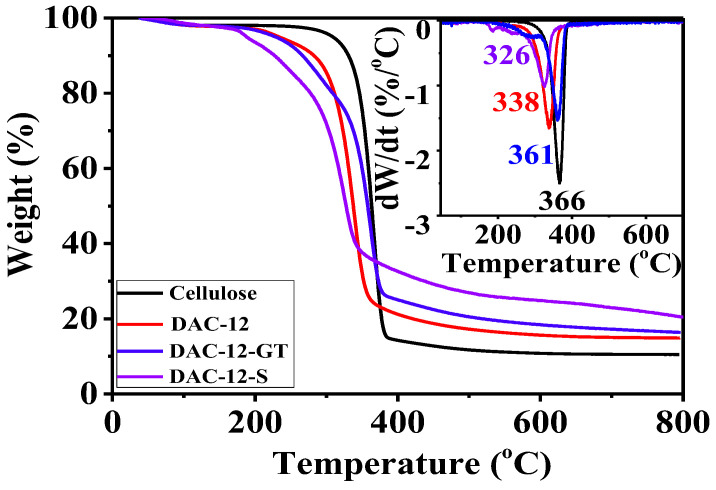
TGA/DTG curves of cellulose membrane before and after modification.

The thermal behavior of the cellulose membrane, DAC-12, DAC-12-GT, and DAC-12-S was investigated, as shown in Figure 8, which reflected the changes in the composition of cellulose after oxidation, quaternization, and sulfonation.

It is seen that the initial decomposition temperature of cellulose membrane is 256 °C, and rapid weight loss occurred at 337~379 °C; while the initial decomposition temperature of modified cellulose decreased, and the decomposition and weight loss began rapidly at 296 °C~355 °C, 296 °C~373 °C and 297 °C~344 °C, respectively [28]. From the DTG curves, the degradation rates of DAC-12, DAC-12-GT, and DAC-12-S decreased during the rapid weight loss stage, while the maximum degradation rates were increased in the order of DAC-12-S, DAC-12, DAC-12-GT, and cellulose, corresponding to 326, 338, 361, and 366 °C, respectively. This is because DAC oxidation destroys the ordered bonding of cellulose [29], thereby decreasing the crystallinity and resulting in a decrease in thermal stability [30] compared to pristine cellulose. Meanwhile, the quaternary and sulfonation reactions introduced non-carbon elements, such as nitrogen and sulfur, which may retard or promote the cracking and carbonization of cellulose to a certain extent.

### 3.4. Mechanical Properties

**Figure 9:**
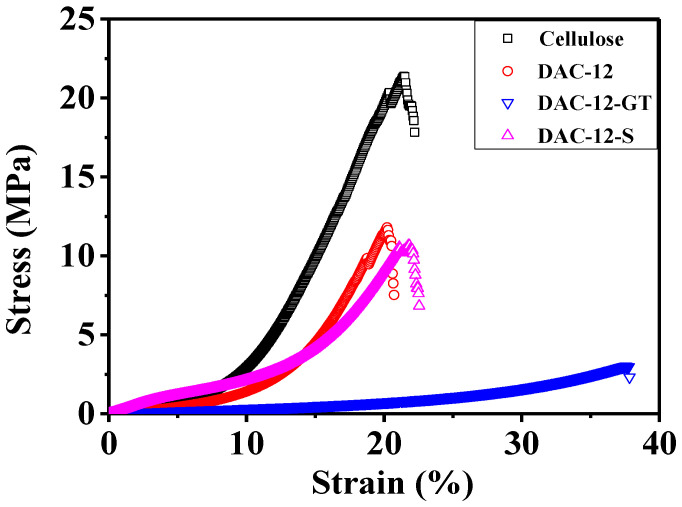
Stress–strain curves of pristine, oxidized, quaternized, and sulfonated cellulose membranes.

The mechanical properties of oxidized, quaternized, and sulfonated cellulose membranes were tested and compared with that of pristine cellulose fabrics, and the results are shown in Figure 9.

It is interesting that the ultimate tensile strength of pristine cellulose fabrics is 21.36 MPa, which drastically decreased to 11.81 MPa after periodate oxidation, probably due to the degradation of cellulose molecular chains through the oxidation reaction [28], though the morphology of the oxidized cellulose membrane remained the same apparently. The tensile strength of DAC-12-S is 10.66 MPa, which is comparable to that of DAC-12 exhibiting reasonably good mechanical properties. The ultimate tensile strength of DAC-12-GT was decreased drastically to 3.01 MPa; however, the elongation-to-break was increased from 22.22% to 37.83%, implying that the quaternized cellulose membrane was more flexible. The decrease in tensile strength of the quaternized cellulose membrane could be attributed to the breaking down of cellulose chains by oxidation and the hydrogen bonding broken after the introduction of quaternary ammonium groups, which agreed with the SEM results. Nevertheless, the quaternized cellulose membrane is strong enough to be used as a filter and is comparable to or even better than other cellulose filters [31,32,33,34].

### 3.5. Hydrophilic Properties

**Figure 10:**
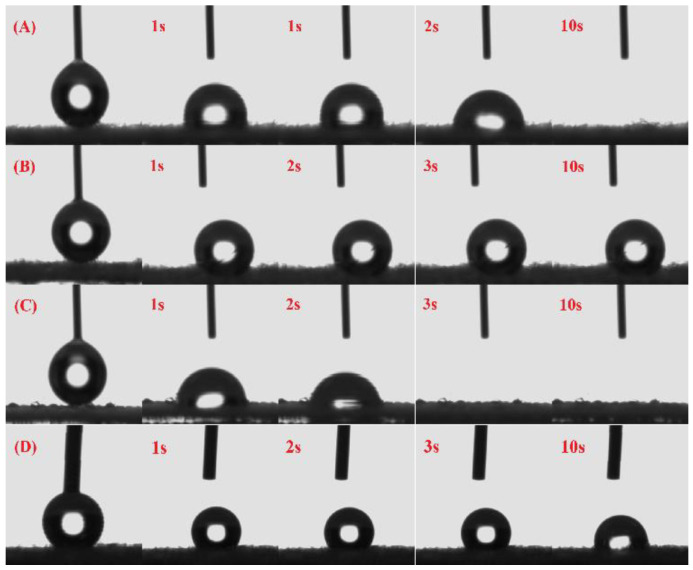
Water contact angles of cellulose membranes vs. time: (**A**) pristine cellulose, (**B**) DAC-12, (**C**) DAC-12-GT, and (**D**) DAC-12-S.

The hydrophilicity of the cellulose membrane was affected drastically after oxidation, quaternization, and sulfonation. Therefore, water contact angle measurements of the samples including pristine, oxidized, and quaternized cellulose fabrics were conducted, as shown in Figure 10.

The water contact angle of the pristine cellulose membrane decreases quickly with time, showing hydrophilic properties, because of many hydrophilic hydroxyl groups on the surface of the cellulose fiber [35]; however, the water contact angle of DAC-12 remained unchanged with time due to high hydrophobic properties. In other words, periodate oxidation converts hydrophilic hydroxyl groups in cellulose into hydrophobic aldehyde groups, which makes the DAC-12 membrane exhibit a hydrophobic nature. Compared with DAC-12, the hydrophilicity of DAC-12-S was improved to a certain extent because part of the hydrophobic aldehyde groups was converted into hydrophilic sulfonate groups. The water contact angle of DAC-12-GT, however, decreased rapidly with time, indicating that the quaternization reaction changed the hydrophilicity of cellulose drastically and even greatly enhanced it. This would be beneficial to the adsorption performance of the quaternized cellulose membrane by improving the wetting ability of water on the cellulose.

### 3.6. Hydrophilic Properties

#### 3.6.1. Effect of Solution pH and Adsorption Time on Adsorption Capacity

**Figure 11:**
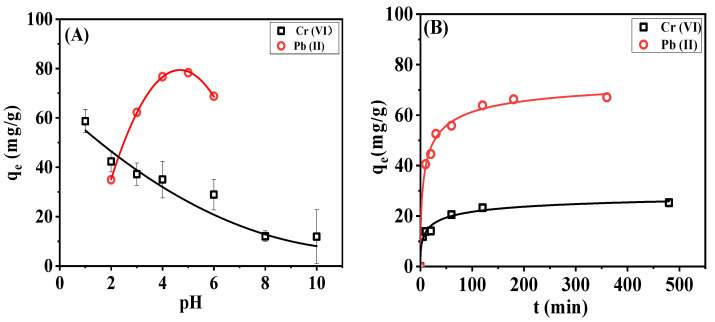
Effects of solution pH values (**A**) and adsorption time (**B**) on the adsorption capacity of DAC-12-GT and DAC-12-S, respectively.

Wastewater containing Cr(VI) and Pb(II) ions could be purified by quaternized and sulfonated cellulose membranes, respectively, through the removal of the heavy metal ions. A series of static adsorption experiments were conducted to determine the optimized pH values and the time to the equilibrium state of the adsorption system, as shown in Figure 11.

It can be seen that the adsorption capacity of DAC-12-GT membrane against Cr(VI) decreased with the increase in pH values, and the highest adsorption capacity of 58.9 mg/g was achieved when the pH value was 1.0. This is probably due to the effects of the electrostatic interaction between the adsorbent and the target with opposite charges, resulting in a decrease in adsorption capacity [14]. The formats of Cr(VI) ions in an aqueous solution are H_2_CrO_4_, HCrO_4_^−^, Cr_2_O_7_^2−^, and CrO_4_^2−^ when the pH value is 2.0, and HCrO_4_^−^ is the dominant species, which promotes the binding of positively charged DAC-12-GT. The adsorption of DAC-12-GT membrane reached equilibrium status rapidly within 3 h, where the equilibrium adsorption capacity was 23.7 mg/g.

With the increase in pH, the adsorption capacity of DAC-12-S membrane for Pb(II) ions exhibited a trend of first increasing and then decreasing. The content of H^+^ in the solution was high when the pH value of the aqueous solution was low, which could have induced a competitive adsorption with Pb(II), leading to a low adsorption capacity of Pb(II) on the sulfonated cellulose membrane. With the increase in pH value, the negative charges on the surface of the cellulose membrane accumulated, and the electrostatic interaction between the adsorption site on the surface of the cellulose membrane and Pb(II) ions was enhanced; as a result, the adsorption capacity of DAC-12-S membrane against Pb(II) increased. However, Pb(II) ions will be in the format of precipitation [36] when the pH value of the system continuously rises to 6 or higher. Therefore, the optimum pH value for Pb(II) ion removal was determined to be about 5, and the maximum removal capacity of DAC-12-S was up to 78.3%.

#### 3.6.2. Adsorption Kinetics

**Figure 12:**
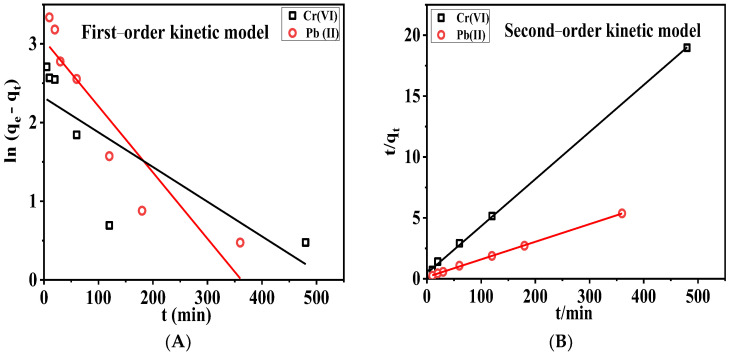
Fitting curves of first-order adsorption kinetic model (**A**) and of second-order adsorption kinetic model (**B**).

The relationship between the adsorption capacity and adsorption time was established by the adsorption kinetics analysis. Two typical kinetic models, pseudo-first-order and pseudo-second-order kinetic models, were employed to fit the data. The results were shown in Figure 12 and Table 1.

The relationship between the adsorption capacity and adsorption time was established by the adsorption kinetics analysis. Two typical kinetic models, pseudo-first-order and pseudo-second-order kinetic models, were employed to fit the data. The results are shown in Figure 12 and Table 1.

It is clear that the adsorption of the cationic/anionic cellulose membranes follows the pseudo-second-order kinetics, with adsorption rate constants of 0.0033 and 0.0015 g·min^−1^·mg^−1^, respectively. Therefore, the adsorption of Cr(VI) and Pb(II) by cationic/anionic cellulose membranes belongs to chemical adsorption, that is, the electrons shared between the quaternary ammonium and Cr(VI), and between the sulfonate and Pb(II), respectively.

#### 3.6.3. Isotherms

The adsorption isotherm reflects the relationship between the initial concentration of the feed solution and the adsorption capacity of the cellulose membrane. The commonly used isotherm adsorption models are Langmuir, Freundlich, Tempkin, and Dubinin–Radushkevich models [37,38,39]. The best isotherm model was selected according to the correlation coefficient, and then the adsorption mechanism of ionized cotton fiber fabric to Cr(VI) and Pb(II) was explored, as shown in Table 2.

It was found that the Freundlich model could better fit the adsorption process of Cr(VI), which indicates that the adsorption mechanism of the quaternized cellulose membrane for Cr(VI) is the coexistence of monolayer adsorption and multimolecular adsorption. In addition, the maximum adsorption capacity of DAC-12-GT membrane for Cr(VI) was 61.73 mg/g, evaluated by the Langmuir model.

As expected, the adsorption of Pb(II) ions on DAC-12-S membrane was more consistent with the Langmuir model, that is, monolayer adsorption, and the adsorption process easily occurred because 0.1 < 1/n = 0.12 < 0.538 [40]. The maximum adsorption capacity of the sulfonated cellulose membrane for Pb(II) was up to 63.69 mg/g.

#### 3.6.4. Adsorption Thermodynamics

**Figure 13:**
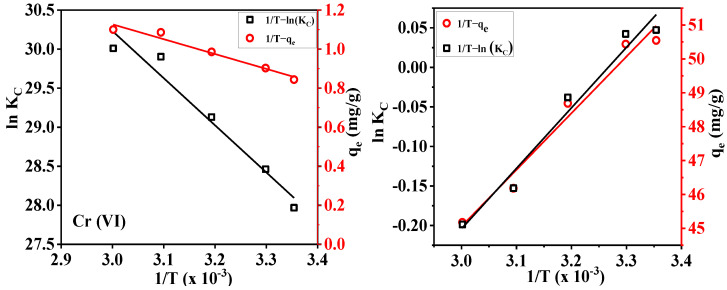
Adsorption thermodynamic profiles of cationic/anionic cellulose membranes.

The thermodynamic behaviors of the cationic/anionic cellulose membranes DAC-12-GT and DAC-12-S were investigated and analyzed, as shown in Figure 13.

The parameters of adsorption thermodynamics were obtained by Van’t Hoff Equations (5)–(7), and the results are depicted in Table 3 [41]:

(5) $$ln\frac{q_{e}}{C_{e}}=\frac{\Delta S}{R}-\frac{\Delta H}{RT}$$

(6) $$K_{C}=\frac{q_{e}}{C_{e}}$$

(7) ΔG=ΔH−TΔS

The thermodynamic equilibrium constant *K_C_* and the adsorption capacity increase with the increase in the temperature, so a high operating temperature is beneficial to the adsorption of Cr(VI) by DAC-12-GT membrane. Moreover, the Gibbs free energy difference ΔG < 0 at room temperature indicates that the adsorption process of cationic/anionic cellulose fabrics for heavy metal ions is spontaneous and thermodynamically favorable [42]. Meanwhile, the effect of temperature on the adsorption capacity was further confirmed by the enthalpy difference ΔH. The entropy difference ΔS > 0 of Cr(VI) indicates that the order of the whole system is reduced, which is because the ion exchange process is divided into two processes: the desorption of the original adsorbed substance and the adsorption of Cr(VI). The decrease in order is attributed to the increase in disorder caused by material desorption [43]. The adsorption capacity of Pb(II) decreased with increasing temperature because the adsorption process was impaired by the high mobility of metal ions at high temperatures, and the tendency of metal ions to escape from the adsorbent surface to the liquid phase increased [44].

The maximum adsorption capacities of the quaternized and sulfonated cellulose membranes DAC-12-GT and DAC-12-S against Cr(VI) and Pb(II), respectively, were compared with other adsorbents, as shown in Table 4 and Table 5.

Compared with other biomass adsorbents [45,46,47,48,49,50,51,52,53,54,55,56,57,58,59,60], the maximum adsorption capacities of DAC-12-GT and DAC-12-S membranes against Cr(VI) and Pb(II) were 61.73 mg/g and 63.69 mg/g, which are comparable or higher, indicating that the highly efficient cationic/anionic cellulose membranes could be used as a candidate for industrial wastewater treatment regarding the removal of Cr(VI) and Pb(II) heavy metal ions.

### 3.7. Dynamic Adsorptions

**Figure 14:**
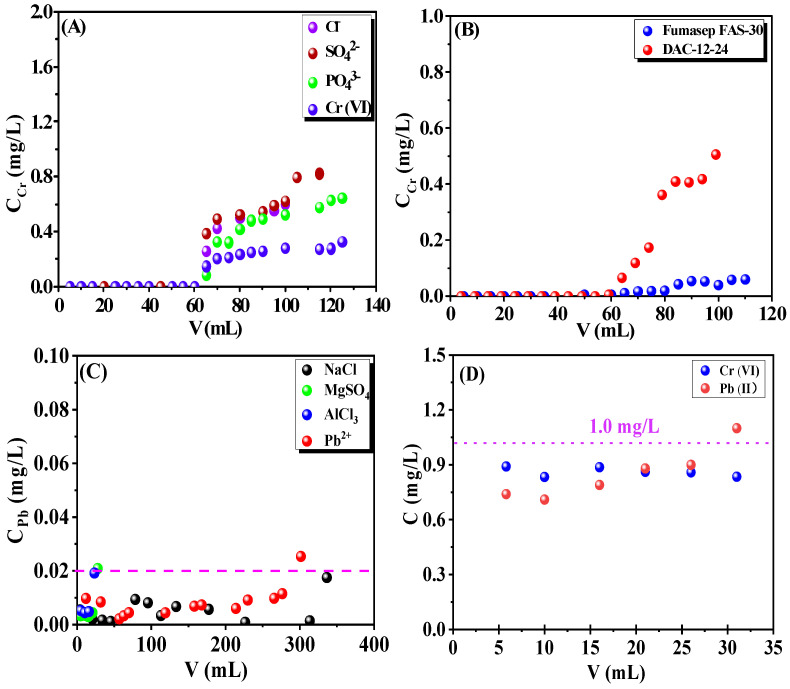
Dynamic adsorption capabilities of the quaternized cellulose membrane DAC-12-GT for Cr(VI) in the contaminated wastewater containing Cl^−^, SO_4_^2−^, and PO_4_^3−^ ions, respectively (**A**); dynamic adsorption capacities of DAC-12-GT and commercially available ion-exchange membranes for Cr(VI) (**B**); dynamic adsorption capacities of DAC-12-S membrane for Pb(II) interfered by 0.5 mg/L NaCl, MgSO_4_, and AlCl_3_, respectively (**C**); and dynamic adsorption capacities of unmodified cellulose membranes for Cr(VI) and Pb(II) at 1.0 mg/L, respectively (**D**).

The dynamic adsorption performance of the cationic/anionic cellulose membranes was demonstrated, where various interfering ions with a five-times higher concentration co-existed in the feeding solution, respectively, and the breakthrough curves in terms of the heavy metal ion concentration of permeate versus treated volume were achieved, as shown in Figure 14.

It can be seen that the DAC-12-GT exhibited a 100% rejection ratio against Cr(VI) when the total volume of 60 mL of the solution was purified completely; however, the unmodified cellulose membrane (Figure 14D) has a rejection ratio of less than 10% for Cr(VI), indicating that the DAC-12-GT membrane could be an efficient filter for removal of Cr(Ⅵ) from wastewater. Meanwhile, the interfering ions including Cl^−^, SO_4_^2−^, and PO_4_^3−^, with five-times higher concentration (5 mg/L) were employed to challenge the adsorption capacity of the quaternized cellulose membrane, as shown in Figure 14A, while the pH value of the feeding solution was 2.0 and competing ions with a five-times higher concentration co-existed. Again, 60 mL more Cr(VI) solution was completely purified with a 100% of rejection ratio, while the flow rate remained at 0.05 mL/min. These results are impressive in that the quaternized cellulose membrane exhibited outstanding selectivity against competing ions for the dynamic adsorption of Cr(VI) under these conditions. This could be attributed to the adsorbent-Cr(VI) structure being more stable due to the electron sharing of quaternary ammonium salts with Cr(VI) [61], although the adsorption of Cr(VI) by a quaternized cellulose membrane is typically based on an ion-exchange mechanism.

A commercially available anion-exchange membrane, Fumasep-FAB-PK130, was employed for the removal of Cr(VI) and compared with DAC-12-GT, as shown in Figure 14B. The rejection ratio of Fumasep-FAB-PK130 was retained at 100% against Cr(VI) ions when 60 mL of wastewater was treated, i.e., the adsorption capability of the DAC-12-GT was comparable with that of Fumasep-FAB-PK130.

Moreover, it can be seen from Figure 14C that DAC-12-S can purify 300 mL of wastewater containing 0.1 mg/L of Pb(II) ions and 0.5 mg/L of Na^+^, Mg^2+^, and Al^3+^ ions when the removal rate of Pb(II) retained 75%. It is clear that multi-covalent ions such as Mg (II) and Al (III) exhibit serious effects on the adsorption capacity of DAC-12-S against Pb(II), while monovalent ions, i.e., Na^+^, have a negligible effect in the removal of Pb(II). 

### 3.8. Evaluation of Long-Term Adsorption Capacity of Spiral Wound Adsorption Cartridge

**Figure 15:**
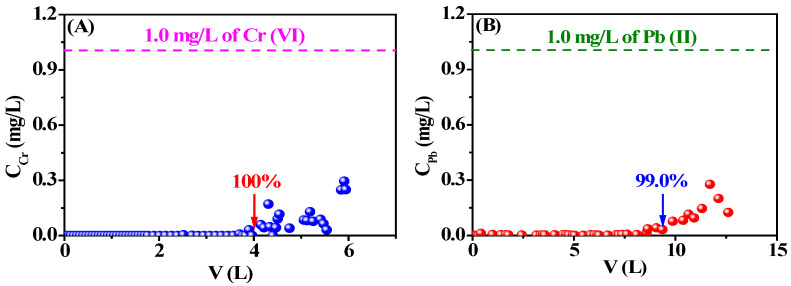

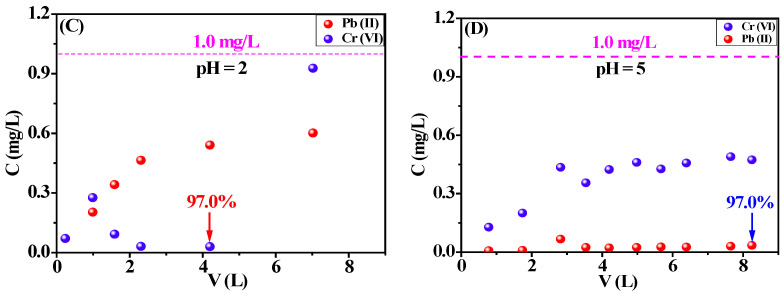
Breakthrough curves of (**A**) DAC-12-GT- and (**B**) DAC-12-S-based adsorption cartridges against Cr(VI) and Pb(II), respectively, and the composite cartridges against Cr(VI) and Pb(II) ions, respectively, at pH = 2 (**C**) and pH = 5 (**D**).

In order to verify the possibility of the practical application of the cationic/anionic cellulose membranes, DAC-12-GT and S-12-24 were employed to manufacture spiral wound adsorption cartridges, and 1.0 mg/L Cr(VI) and Pb(II) aqueous solutions were prepared for the long-term demonstration. The flow rate was 1.0 mL/min, while the pH values remained at 2.0 and 5.0, respectively. The breakthrough curve is shown in Figure 15.

The dynamic adsorption of the DAC-12-GT and DAC-12-S membrane-based spiral wound adsorption cartridges against Cr(VI) and Pb(II), respectively, was conducted to demonstrate the adsorption capability of the cartridge for the long-term purification process. A 100% rejection ratio was achieved when about 4.0 L of Cr(VI) aqueous solution was purified, and 5g of the DAC-12-GT membrane could be used for about 3 days, which indicates the DAC-12-GT membrane would be appropriate for potential commercial applications. Meanwhile, the DAC-12-S membrane-based spiral wound adsorption cartridge exhibited retention of 97.0% in 6 days when 8.6 L of feeding aqueous solution containing Pb(II) was employed. The pressure drops of Cr(VI) and Pb(II) in the systems were retained at 246 Pa and 234 Pa, respectively. 

**Figure 16:**
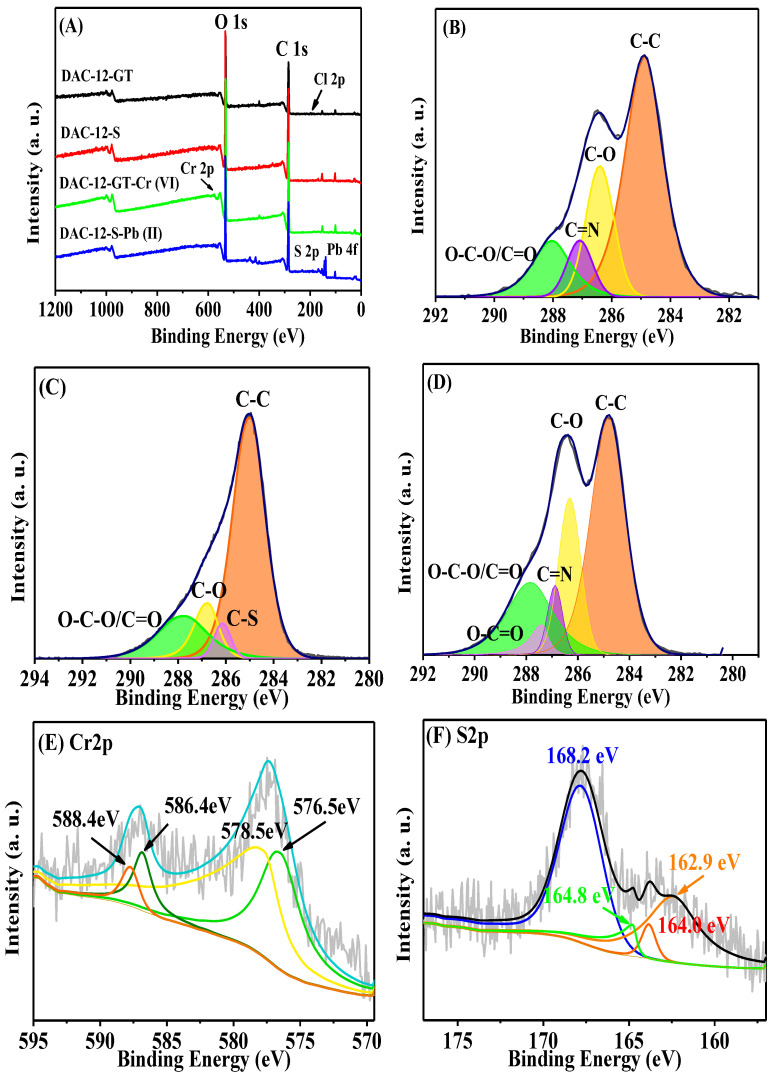
Surface XPS wide-scan spectra of DAC-12-GT and DAC-12-S membranes before and after adsorption (**A**) and C1s high-resolution XPS energy spectra of DAC-12-GT (**B**), DAC-12-S (**C**), DAC-12-GT-Cr(VI), C1s (**D**), DAC-12-GT-Cr(VI), Cr2p (**E**), and DAC-12-S-Pb(II), S2p (**F**).

In addition, a composite cartridge was fabricated by the integration of DAC-12-GT and DAC-12-S membranes and was employed to adsorb both Cr(VI) and Pb(II) ions simultaneously, as shown in Figure 16C,D, respectively. It was very interesting that the retention rate of Cr(VI) was higher than 97.0% when 4.2 L of the feed solution containing Cr(VI) and Pb(II) ions at pH 2.0 was purified. However, the retention of Pb(II) ions was decreased to 46.0% due to the acidic feed solution. When the pH value of the feed solution was adjusted to 5.0, the retention rate of Cr(VI) retained at 53.0%, though the retention rate of Pb(II) was as high as 97.0% when 8.3 L of wastewater was treated, as shown in Figure 15D. Therefore, the integrated cationic/anionic cellulose membranes-based adsorption cartridge could be potentially used for the purification of industrial wastewater containing Cr(VI), Pb(II), as well as other heavy metal ions.

### 3.9. Adsorption Mechanism

The adsorption mechanism of quaternized and sulfonated cellulose membranes for Cr(VI) and Pb(II) ions, respectively, was proposed based on the investigation of XPS measurements.

Figure 16A shows the XPS wide-scanning spectra of cellulose-based adsorption membranes before and after adsorption, where significant peaks appear near 286.0 eV and 533.1 eV, and these peaks correspond to the energy spectra of C1s and O1s, respectively. In the spectrum of DAC-12-GT-Cr(VI), the new peak at 577.0 eV corresponding to Cr 2p was observed [62], and the peaks located at 140.0 eV and 168.0 eV, respectively, were assigned to Pb 4f and S2p, respectively, indicating that successful adsorption of Cr(VI) and Pb(II) ions occurred. 

The content of Cl decreased from 0.95% to 0.13%, as shown in Figure 16A, indicating that the removal of Cr(VI) ions by DAC-12-GT was carried out by anion exchange with Cl^−^. Moreover, the Cr 2p XPS spectrum showed four peaks, corresponding to that of Cr(VI) $2p^{\frac{1}{2}}$ (588.4 eV), Cr(VI) 2$p^{\frac{3}{2}}$ (578.5 eV), Cr(III) $2p^{\frac{1}{2}}$ (586.4 eV), and Cr(III) 2$p^{\frac{3}{2}}$ (576.5 eV) [24], respectively. The appearance of Cr(III) on the surface of DAC-12-GT implies that a reduction reaction occurred in the adsorption process [24]. According to the O-C=O at 287.4 eV (in Figure 16D, C1s), it can be concluded that the aldehyde group could be further oxidized and the carboxyl group might be generated [25,63,64,65].

In the spectrum of S2p, the peaks of S $p^{\frac{1}{2}}$ and S $2p^{\frac{5}{2}}$ correspond to 168.2 eV and 164 eV, respectively [66]. After the adsorption of Pb(II) ions, the peaks of 162.9 eV and 174.8 eV in the spectrum of DAC-12-S-Pb(II) can be attributed to Pb-S, confirming that Pb(II) ions were successfully adsorbed by sulfonated cellulose and the complex of (R-SO_3_^−^)_2_-Pb^2+^ could be formed, which was achieved by electrostatic interaction of negatively charged sulfonate groups and Pb(II) ions on the sulfonated cellulose membrane [67,68,69].

Based on the above analysis, it was concluded that the adsorption process follows the anion exchange mechanism. Cr(VI) ions were adsorbed on the quaternized cellulose membrane by Cl^−^ ion exchanging and then the adsorbed Cr(VI) ions were partially reduced to Cr(III), while aldehyde groups were oxidized to carboxyl groups. The adsorption of Pb(II) ions follows the electrostatic interaction, while SO_3_^−^ and Pb(II) could form a cation–sulfonate complex. 

**Figure 17:**
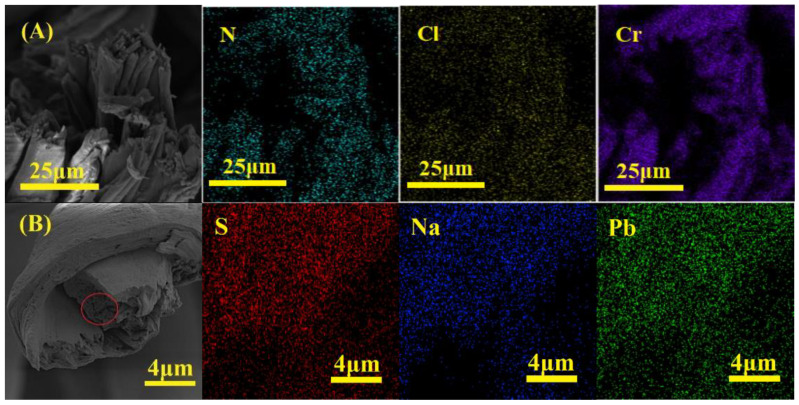
EDS mapping images of the cross-section of DAC-12-GT-Cr(VI) (**A**) and DAC-12-S-Pb(II) (**B**).

EDS mapping provides the distribution view of various elements on the surface of the membrane before and after adsorption, as shown in Figure 17.

The presence of N and S elements was observed in the cross-sectional views of DAC-12-GT and DAC-12-S, respectively, indicating that the quaternization and sulfonation of cellulose occurred not only on the surface but also the inside of the cellulose fibers, i.e., there were adsorption sites located in the fiber inside, which greatly enhanced the adsorption capacity of the membrane. Moreover, the distribution of Cr(VI) and Pb(II) was also found at the cross-section of cellulose fibers after adsorption, which further proves that the adsorption capacity of Cr(VI) and Pb(II) was available on both the surface and the inside of the membrane.

It is worth noting that there were still some chloride and sodium ions anchored on the cellulose fibers, as shown in Figure 17A,B, which implies that the functional groups such as ammonium and sulfonate were only partially used in the adsorption process. Nevertheless, the distribution of nitrogen and sulfur elements on the cross-section also further proves that the porous structure inside the cellulose fibers (as shown in the red circle in Figure 17B) could be used for improving the absorption capacity, resulting in the maximum adsorption capacities of modified cellulose membranes against Cr(VI) and Pb(II) of 61.73 and 63.69 mg/g, respectively.

### 3.10. Desorption

The recycling and reusability of the modified cellulose membranes, either quaternized or sulfonated, were evaluated through desorption experiments, which were carried out by different desorption methods [70,71]. The results are summarized as follows.

It can be seen from Table 6 that the desorption rate of the cationic cellulose membrane against Cr(VI) ions was 13.3% when 1.0 wt% of NaOH aqueous solution was used as the desorption agent. Even enhanced by adding 1.0 wt% of NaCl solution or by increasing the concentration of NaOH, however, the desorption rate still failed to be improved. However, the desorption rate of a commercially available anion-exchange resin containing ammonium groups could reach up to 59.7%, which indicates that the adsorption mechanism of Cr(VI) ions on the quaternized cellulose membrane was more complex than the single ion-exchange interaction and is also consistent with the fact that the adsorption was not affected by coexisting ions.

Moreover, the desorption rates could be reasonably high when 1.0 mol/L HNO_3_ or 1.0 mol/L EDTA solution was employed to desorb Pb(II) from the sulfonated cellulose membrane, which were 45.4% and 61.3%, respectively. This agreed with the ion-exchange adsorption mechanism for the adsorption of sulfonated cellulose membrane against Pb(II) ions.

## 4. Conclusions

Highly efficient cationic/anionic cellulose membranes were fabricated successfully for the removal of Cr(VI) and Pb(II) with a high permeation flux and a low-pressure drop. In addition to comprehensive structural and morphological characterizations, the excellent properties of thermal, mechanical, and surface hydrophilicity of the modified cellulose membranes were also investigated and verified. The characterizations and analytical titrations confirmed that the cellulose membrane successfully grafted with quaternized ammonium and sulfonate groups. The adsorption capability of the quaternized and sulfonated cellulose membranes against Cr(VI) and Pb(II) was determined and analyzed, while the maximum adsorption capacity was 61.7 and 63.7 mg/g, respectively, which was better than that of most biomass-based adsorbents. The adsorption mechanism of the cellulose membranes was proposed based on electrostatic interaction and ion exchange, while a reduction reaction could have occurred in the adsorption process of Cr(VI). The dynamic adsorption capabilities of the cationic/anionic membranes were also investigated from the aspects of dynamic adsorption when competitive ions coexisted. The competing ions exhibited less effect on the adsorption of Cr(VI) by the quaternized cellulose membrane even with five-times higher concentration; however, the adsorption capability of Pb(II) by the sulfonated cellulose membrane was significant when multi-valent ions were co-existing. Spiral wound adsorption cartridges based on quaternized and sulfonated cellulose membranes were fabricated and used to challenge Cr(VI) and Pb(II) aqueous solutions for long-term adsorption. The quaternized cellulose membrane-based adsorption cartridge shows a 100% high retention rate when 4.0 L of Cr(VI)-containing wastewater was purified, and the pressure drop was as low as 246 Pa. Meanwhile, the sulfonated cellulose membrane-based cartridge can adsorb 8.6 L of Pb(II) ion solution, while the retention rate was retained at 99%, and the pressure drop was 234 Pa. Interestingly, the integrated cartridge prepared by the combination of cationic and anionic cellulose membranes also exhibited high retention rates for both Cr(VI) and Pb(II) in the treatment of wastewater containing Cr(VI) and Pb(II) ions simultaneously, while retention rate of the composite cartridge was as high as 97.0%, achieved for both Cr(VI) and Pb(II) at the optimized pH values. Therefore, the removal efficiency of Cr(VI) and Pb(II) ions by the cationic/anionic cellulose membranes issued significant advantages over other counterparts, which certainly suggests that the cationic/anionic cellulose membranes could be potentially used for large-scale wastewater treatments.

## Figures and Tables

**Table 1:** Kinetic parameters of adsorption of Cr(VI) and Pb(II).

First-Order Kinetic Model $ln\;\left(q_{e}-q_{t}\right)=lnq_{e}-K_{1}t$				Second-Order Kinetic Model $\frac{t}{q_{t}}=\frac{1}{K_{2}\times q_{e}^{2}}+\frac{t}{q_{e}}$		
	*q_e_* (mg/g)	*K*_1_ (min^−1^)	*R* ^2^	*q_e_* (mg/g)	*K*_2_ (g·min^−1^·mg^−1^)	*R* ^2^
Cr(VI)	10.14	0.0044	0.6613	25.84	0.0033	0.9995
Pb(II)	66.27	0.0084	0.8608	68.97	0.0015	0.9996

**Table 2:** Fitting parameters of isotherm adsorption models.

Model Type	Model Equations	Parameters	
Langmuir	$\frac{1}{q_{e}}=\frac{1}{q_{m}\times K_{L}}\times \frac{1}{C_{e}}+\frac{1}{q_{m}};$ $\frac{1}{q_{e}}=\frac{1}{q_{m}\times K_{L}}\times \frac{1}{C_{e}}+\frac{1}{q_{m}};$ $R_{L}=\frac{1}{1+K_{L}q_{m}}$	*R* ^2^	0.98
			0.97
		*q_m_*	61.73
			63.69
		*K_L_*	0.0038
			0.083
		*R_L_*	0.81
			0.16
Freundlich	$loglog\;\left(q_{e}\right)=\frac{1}{n}loglog\;\left(C_{e}\right)\;$ $+log\left(K_{F}\right)$	*R* ^2^	0.98
			0.96
		*n*	1.40
			8.33
		*K_F_*	0.56
			17.38
Tempkin	$q_{e}=\frac{RT}{b_{T}}\times lnln\;\left(C_{e}\right)\;$ $+\frac{RT}{b_{T}}\times ln\left(K_{T}\right)$	*R* ^2^	0.90
			0.96
		*b_T_*	158.76
			200.88
		*K_T_*	0.031
			1.22
Dubinin–Radushkevich	$lnq_{e}=lnq_{m}-K_{D}B^{2};$ $B=RTln\left(1+\frac{1}{C_{e}}\right);$ $E_{s}=\frac{1}{\sqrt{2K_{D}}}$	*R* ^2^	0.83
			0.90
		*q_m_*	30.68
			54.60
		*K_D_*	8 × 10^−7^
			1.75 × 10^−5^
		*E_S_*	790.51
			168.92

**Table 3:** Adsorption thermodynamic parameters.

					Δ*G* KJ/mol				
	*R* ^2^		Δ*H* KJ/mol	Δ*S* J/(mol·K)	*T*/°C				
	1/*T* − *q_e_*	1/*T* − ln(*K_C_*)			25	30	40	50	60
Cr(VI)	0.96	0.96	6.32	28.34	−2.13	−2.27	−2.55	−2.84	−3.12
Pb(II)	0.97	0.97	−5.95	−19.54	−0.13	−0.03	0.16	0.36	0.55

**Table 4:** Comparison of Cr(VI) and Pb(II) adsorption capacities of different adsorbents.

Adsorbents	pH	*q_m_* (mg/g)
Polyethyleneimine modified ethyl cellulose	1.0	36.8
Polyaniline coated ethyl cellulose	1.0	38.8
Apple peel	2.0	36.0
Sunflower scrap	2.0	53.8
EDTA-modified sisal natural fiber	2.0	61.5
Banana Biochar	2.0	125.4
Grapefruit peel biochar	2.0	57.7
Aminated cross-linked chitosan	2.0	352.0
Wheat bran	3.0	0.9
Walnut shells	3.0	50.1
Chitosan electrospinning fiber	3.0	208.0
Coconut fiber	4.5	4.6
Corn stalks	4.5	89.5
Activated carbon supported amine cross-linked copolymer	5.0	102.9
Cationic cross-linked starch	5.5	74.4
This work	2.0	61.7

**Table 5:** Comparison of Pb(II) adsorption capacity of different adsorbents.

Adsorbents	pH	*q_m_* (mg/g)
Mesoporous activated carbon	7	20.3
Cellulose-chitosan–pyridine	5	80.3
Thiol-modified rice straw biochar	5	61.4
Thiol-functionalized cellulose nanofiber	4	22.0
Apple pulp activated carbon	5.5	15.96
Magnetic litchi peel	6	78.7
Cellulose acetate electrospinning membrane	6	70.50
Biochar derived from poplar saw dust	5	62.68
Chitosan microspheres	7	78.9
Phosphoric acid-based cellulose microspheres	5	108.5
This work	5	63.7

**Table 6:** Desorption efficiency of cationic/anionic cellulose membranes for Cr(VI) and Pb(II) via different desorption approaches.

Desorption Agent	Adsorbent	Desorption Rate (%)
1.0 wt% NaOH solution	DAC-12-GT	13.3
Mixture of 1.0 wt% NaOH and 1.0 wt% NaCl solutions	DAC-12-GT	12.5
10 wt% NaOH solution	DAC-12-GT	4.8
1.0 mol/L HNO_3_	DAC-12-S	45.4
1.0 mol/L EDTA	DAC-12-S	61.3
